# Nanostructured morphology of a random P(DLLA-*co*-CL) copolymer

**DOI:** 10.1186/1556-276X-7-103

**Published:** 2012-02-05

**Authors:** Laura Peponi, Angel Marcos-Fernández, José María Kenny

**Affiliations:** 1 Instituto de Ciencia y Tecnología de Polímeros, ICTP-CSIC, Juan de la Cierva, 3 Madrid 28006, Spain

**Keywords:** nanostructutation, random copolymer, biomaterials, polylactic acid, poly(ε-caprolactone)

## Abstract

The random architecture of a commercial copolymer of poly(DL-lactic acid) and poly(ε-caprolactone), poly(DL-lactide-*co*-caprolactone), has been characterized by chemical structure analysis from hydrogen-1 nuclear magnetic resonance results. Moreover, spherical nanodomains have been detected in the thin films of this copolymer obtained after solvent evaporation. These nanodomains studied by atomic force microscopy and transmission elecron microscopy grow progressively under annealing until they collapse and form a homogenous disordered structure. This is the first time that the nanostructure of random poly(DL-lactic acid)/poly-(ε-caprolactone) copolymers is revealed, representing one of few experimental evidences on the possible nanostructuration of random copolymers.

## Background

In the past years, the request of polymers for applications in the biomedical sector has grown drastically. Among others, poly-(lactic acid) [PLA], derived from renewable resources, is currently being used in a number of biomedical applications, such as in sutures, stents, drug delivery devices, and tissue engineering [1]. Besides, the biodegradable petroleum-based polyester poly-(ε-caprolactone) [PCL] has also been widely studied [2-4]. However, these polymers are inappropriate for numerous uses where highly flexible biodegradable materials are required [5]. Therefore, different strategies have been reported to properly modify the intrinsic properties of both polymers, including the use of additives and nanoparticles [6-8]. Another possible strategy is constituted by blending or copolymerizing them together, allowing the fabrication of a variety of biodegradable materials with improved properties in comparison with those of the parent homopolymers [3,9]. Biodegradable PLA-blend-PCL materials can offer a wide variety of physical properties; the glassy PLA with a relatively high degradation rate shows better tensile strength, while the rubbery PCL with a much slower degradation rate shows better toughness [10]. As reported in the scientific literature, the PCL/PLA blend can form typical immiscible morphologies (of few micrometer scales) such as spherical droplets, fibrous and co-continuous structures by varying the homopolymer composition [11,12].

In the general case of copolymers, their final properties depend not only on their composition but also on their architecture (i.e., random, alternate, or block). Random and alternate copolymers are reported to be typically one-phase disordered materials with concentration fluctuations of a relatively short range [13]. On the other hand, block copolymers present phase separation in the nanometer range, taking advantage of the covalent bonding between the immiscible constituting blocks which are able to self-assemble into well-defined ordered nanostructures with domain dimensions of 5 to 100 nm [14-17]. It is, therefore, not surprising that block copolymers have attracted worldwide attention of physicists, chemists, and engineers, developing numerous applications ranging from thermoplastic elastomers, adhesives, sealants, polymer blend compatibilizers, emulsifiers, and other recent advances in their medical applications [17-21]. The main features of these nanostructures, such as their composition, morphology, dimensions, spacing, and order are of primary significance for the chemical, mechanical, optical, and electromagnetic properties exhibited [22,23].

Few studies have been reported on PLA/PCL copolymers with particular focus on their crystallization behavior [3,5]. In this work, a commercial random copolymer based on poly(DL-lactic acid) [PDLLA] and PCL is studied, focusing the attention on its chemical architecture and nanostructured morphology.

## Methods

### Materials

The copolymer based on poly(DL-lactic acid) and poly(ε-caprolactone), poly(DL-lactide-*co*-caprolactone) [P(DLLA-*co*-CL)], was supplied by Sigma Aldrich (St. Louis, MO, USA) with a nominal 86 mol% of PDLLA. Their solubility parameters calculated based on the Hoftyzer-van Krevelen theory [24] are 27 and 25, respectively. Pure chloroform from Sigma Aldrich was used as solvent.

### Sample preparation

A solution of 0.01 g of P(DLLA-*co*-CL) in 5 mL of chloroform has been obtained by stirring the sample for 12 h at room temperature in a closed vessel.

### Physicochemical analysis

The copolymer was characterized by hydrogen-1 nuclear magnetic resonance [1H-NMR] in a Varian Mercury 400 apparatus (Varian Inc., Palo Alto, CA, USA) at 400 MHz, using CDCl_3 _as solvent, and by a relaxation time between pulses of 5 s. The residual signal of the deuterated solvent was used as the internal reference (7.26 ppm).

Raman spectra were obtained using a Renishaw *in via *Reflex Raman System (Renishaw plc, Wotton-under-Edge, UK) employing a laser wavelength of 785 nm (laser power at sample = 10 mW; microscope objective = × 100). Spectra were recorded at room temperature after the exposure time of 10 s, which is necessary to decay the fluorescence.

### Morphological analysis

The morphological features of the copolymer films were investigated using atomic force microscopy [AFM] and transmission electron microscopy [TEM]. The AFM is operating in a tapping mode [TM] with a scanning probe microscope (Nanoscope IV, Multimode TM from Veeco-Digital Instruments, Plainview, NY, USA). Height and phase images were obtained under ambient conditions with a typical scan speed of 0.5 to 1 line/s, using a scan head with a maximum range of 100 μm × 100 μm. The TEM measurements were performed on a JEOL JEM-2100 TEM instrument (JEOL Ltd., Akishima, Tokyo, Japan), with a LaB6 filament, with an operating voltage of 200 kV. For the morphological analysis by atomic force microscopy, a transparent thin film (*ca*. 300 nm) was obtained using a spin coater SCS P-6700 (Special Coating Systems, Inc., Indianapolis, IN, USA) at 4,000 rpm for 140 s followed by solvent evaporation at ambient conditions for 24 h, while for the TEM analysis, the solution, which was twice diluted, has been cast directly on the grid and evaporated at the same conditions.

### Thermal analysis

Differential scanning calorimetry [DSC] measurements were performed with a Mettler-Toledo DSC-822 calorimeter (Mettler-Toledo, Inc., Columbus, OH, USA) calibrated with high-purity indium. All experiments were conducted under a nitrogen flow of 20 mL·min^-1^, using 7- to 10-mg samples in closed aluminum pans, in a temperature range from -90°C to 200°C with a rate of 10°C min^-1^, using a heating-cooling-heating cycle. The second heating scan was used to calculate the glass transition temperature [*T*_g_] of the matrix.

Small-angle X-ray scattering [SAXS] measurements were taken at beamline BM16 at the European Synchrotron Radiation Facility (Grenoble, France). Samples were placed in between aluminum foils within a Linkam hot stage (Linkam Scientific Instruments, Tadworth, UK) and heated at 10°C min^-1 ^while the SAXS spectra were recorded. Calibration of temperature gave a difference of approximately 7°C between the temperature reading at the hot stage display and the real temperature at the sample.

## Results and discussion

The main results on the physicochemical and thermal behaviors of the analyzed random copolymer P(DLLA-*co*-CL) have been the number average molecular weight [*M*_n_] calculated by 1H-NMR and *T*_g _obtained by DSC. In particular, the *M*_n _was calculated through two different approaches: the first one, taking into account the terminal groups, produced a value of 21,000 g/mol, while the ratio between the caprolactone [CL] units and the initiator produced a value of 28,000 g/mol. From thermal analysis, we obtained a *T*_g _of about 24°C.

Moreover, the measured PDLLA content obtained from 1H-NMR was 89.8 mol% in contrast with the value of PDLLA which was 86 mol% given by the supplier. This difference can be considered a small one and in the range of the possible deviation in different batches. In fact, the ratio of the lactic acid [LA] and CL signals allows a quite high accuracy for this calculation with a dispersion of 0.3% in three repetitions. We think that the supplier has provided an average value that could change from batch to batch without reporting the exact value for each batch.

**Figure 1:**
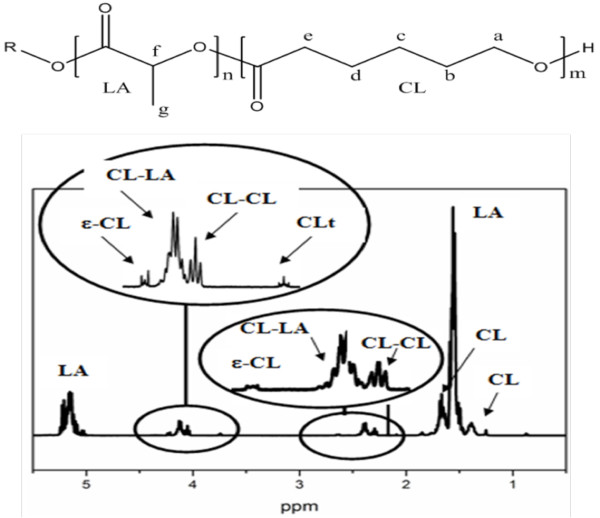
**1H-NMR spectrum of the P(DLLA-*co*-CL) used in this work**. CL refers to polymerized caprolactone units, and LA, to polymerized lactide units. The general chemical structure of the copolymer P(DLLA-*co*-CL) is reported on top (R = polymerization initiator).

The 1H-NMR spectrum for P(DLLA-*co*-CL) is reported in Figure 1, where also the general chemical structure for this copolymer, assuming monofunctional initiator R, is described. This scheme does not imply a di-block structure. The analysis of the 1H-NMR spectrum was performed using Kasperczyk's work as reference [25]. Characteristic signals for polymerized caprolactone and polymerized lactide are observed. The multiplet from 5.05 to 5.25 ppm is assigned to methine proton of polymerized lactide (*f*), with some rests of unpolymerized lactide at approximately 5.03 ppm. Almost undetectable, a negligible signal at approximately 4.35 ppm for terminal LA units appears. At approximately 4.23 ppm and 2.63 ppm, two small signals are due to unreacted ε-caprolactone. Calculations allow the determination of the amount of unreacted ε-caprolactone and unreacted lactide as less than 0.6 wt.% and less than 0.2 wt.%, respectively. The multiplet from 4.08 to 4.18 ppm is due to the CL proton *a *that linked to a LA molecule, while the triplet at 4.05 ppm indicates that the CL proton *a *linked to another CL molecule. The triplet at 3.74 ppm is related to the CL proton *a *for terminal CL molecules (-CH_2_-OH). The multiplet between 2.34 to 2.44 ppm is due to the CL proton *e *that linked to a LA molecule, while the triplet at 2.30 indicates that the CL proton *e *linked to another CL molecule. For the rest of the spectrum, multiplets at 1.66 ppm and 1.39 ppm are related to the CL protons *b, d*, and *c*, respectively, and the multiplet at 1.56 ppm, to the LA methyl protons *g*. So, the ratio of the LA signals to the CL signals results in a molar composition LA/CL of the copolymer of 89.8:10.2 mol% (corresponding to 84.8:15.2 wt.%).

If the copolymer is a di-block copolymer, the ratio of the signal of polymerized CL linked to LA molecules to the signal of terminal CL should be 1, and in our case, it is approximately 8.9. Furthermore, the ratio of the signal due to CL linked to LA to the signal of CL linked to CL is approximately 3.15, indicating the preponderance of isolated CL units in the polymer backbone. From these results, it is clear that the chemical structure of the copolymers approaches more likely the structure of a random copolymer. As the molar content of CL in the copolymer is low, 10.2%, it is reasonable to presume that CL units are isolated in between PLA units (-LA-CL-LA-) or form blocks of double CL units (-LA-CL-CL-LA-), with the existence of longer CL blocks being negligible. Then, from the signals due to CL linked to LA and to CL linked to CL, a 68 mol% of isolated CL units and 32 mol% of double CL units are calculated. Once the total CL and CL-CL units are determined, it is possible to calculate the mean length of the LA blocks which results to 12.

Summarizing the 1H-NMR results, the P(DLLA-*co*-CL) copolymer used in this study has the structure of a predominantly random copolymer with most of the CL units isolated in the copolymer backbone, therefore causing its inability to crystallize, and with blocks of polymerized LA units that are also unable to crystallize, producing an amorphous copolymer. Neither melting nor crystallization was found in the DSC thermogram (not shown), indicating the amorphous nature of the copolymer. The amorphous structure was also confirmed by SAXS (data not shown).

**Figure 2:**
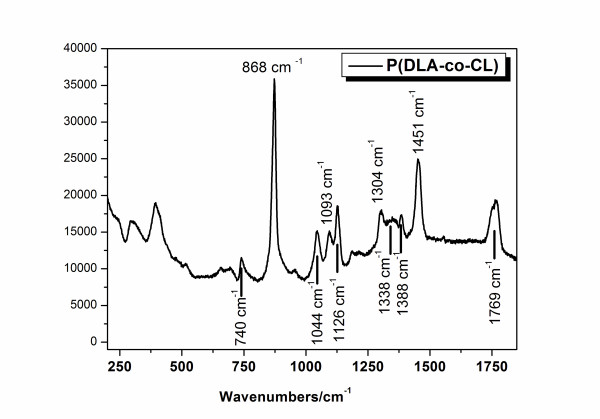
**Raman spectrogram of P(DLLA-*co*-CL)**.

The amorphous state of the copolymer is confirmed also by Raman spectroscopy. In fact, as reported by Kirster et al. [26], the presence of a broad band at 868 cm^-1 ^and the absence of the 1,107-cm^-1 ^and 912-cm^-1 ^narrow peaks are discriminant to characterize the amorphous state of PCL. The spectrogram reported in Figure 2 is in good agreement with this analysis. Above the Raman spectrogram, the values of the main peaks for the CL monomer are indicated, while below the line, the main characteristic peaks for the DLLA monomer are reported. In our case, the Raman line at 868 cm^-1 ^is clearly detected and the peaks at 1107 cm^-1 ^and 912 cm^-1 ^are not detected. Moreover, the large band in the region from 1,300 to 1,360 cm^-1 ^including the Raman line at 1,338 cm^-1 ^confirms the presence of DLLA units and so the amorphous state of the copolymer.

**Figure 3:**
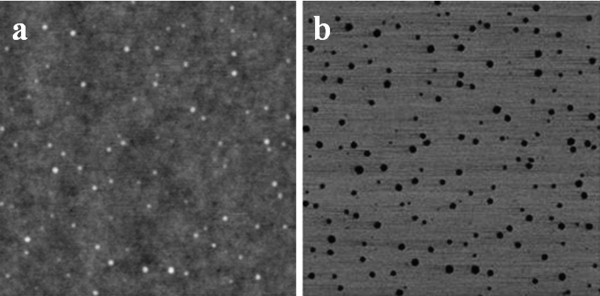
**Images of the solvent spin-cast copolymer film at room temperature**. (**a**) 3 × 3-μm TM-AFM height and (**b**) phase images.

When the copolymer was spin-cast in a film from a chloroform solution, an unexpected nanostructured phase separation was obtained. In fact, the AFM results reported in Figure 3 indicate the formation of a nanostructure with spherical domains having an average diameter of 48 nm.

The nanostructuration observed is coherent with the computer simulation of the phase diagram of random copolymers carried out by Houdayer and Muller [27]. Based on our knowledge, this is the first time that the nanostructuration of a P(DLLA-*co*-CL) copolymer is reported. Moreover, very few experimental demonstrations of the nanostructuration of random copolymers have been reported in the scientific literature [28,29]. Taking into account the small amount of PCL, less than 15 mol%, it is assumed that spherical CL-enriched domains have been obtained. In this case, we consider that, because of the chemical nature of the copolymer, the higher affinity of chloroform for CL than for LA (as obtained by the solubility parameters calculated by the Hoftyzer and van Krevelen theory [24]) has favored the phase separation of CL-enriched domains in a matrix of pure LA or of LA with a lower CL content.

**Figure 4:**
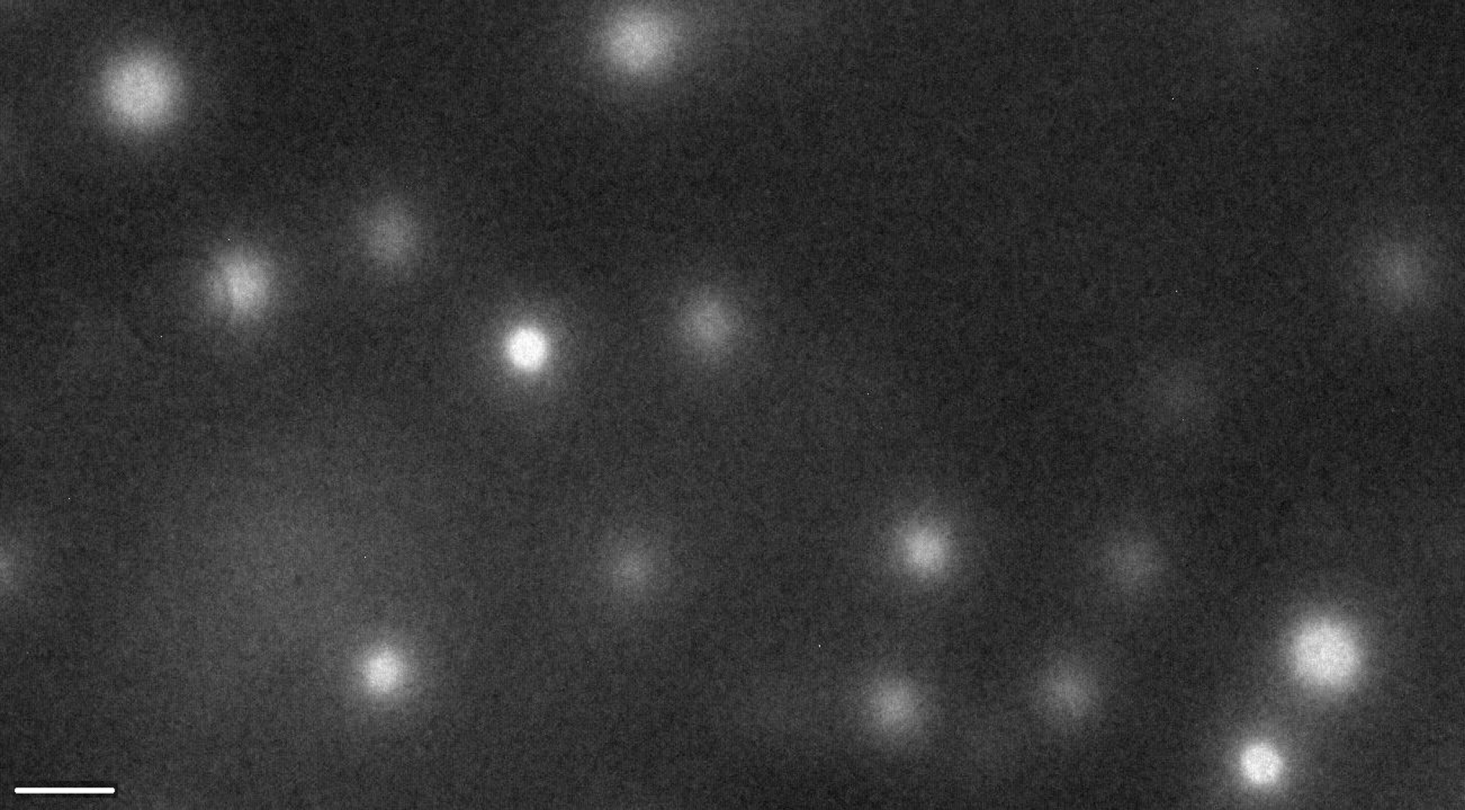
**TEM images of the solvent cast copolymer film at room temperature**. Scale bar, 500 nm.

The same spherical morphology has been detected by TEM analysis, as shown on Figure 4, where spheres with a diameter of about 120 nm are observed. Whereas the TEM image distinguishes between areas with different chemical composition, the AFM image distinguishes between areas with differences in rigidity, leading to the different size determined by both techniques. Moreover, it is known that nanostructuration is strongly dependent on the substrate type and film thickness [23]. In this particular case, the TEM analysis was performed on a 150-nm-thick film on a carbon substrate, while the AFM analysis was performed on a 300-nm-thick film on a glass substrate.

**Figure 5:**
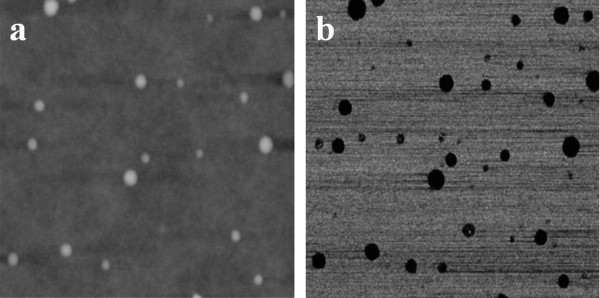
**Images of the 3-h-annealed copolymer at 65°C**. (**a**) 3 × 3-μm TM-AFM height and (**b**) phase images.

After 3 h of annealing treatment at 65°C under vacuum, the spherical domains increased their dimensions (Figure 5), while the fraction of the spherical domains, calculated from the AFM images, remain almost constant (*ca*. 7%). In this case, the spherical domains present an average diameter of 76 nm which is clearly higher than the average diameter of the CL-enriched domains obtained before annealing. This fact indicates that the spherical morphology obtained at an ambient condition represents a non-equilibrium nanostructure that is able to modify itself when the diffusion process is activated by an annealing treatment. This means that the morphological structure obtained is able to reach a free-energy minimization, resulting in the formation of ordered structures as in the case of block copolymers [30].

**Figure 6:**
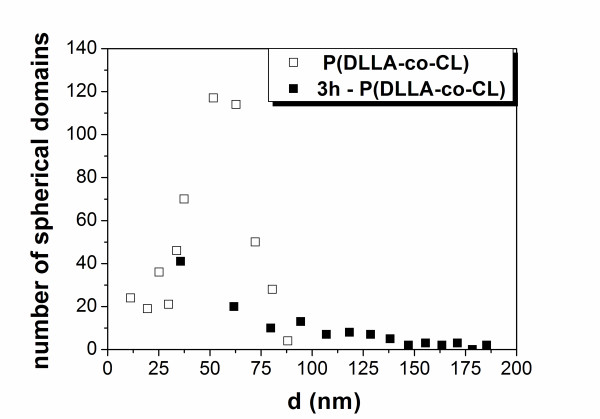
**Statistical distribution**. Statistical distribution of the spherical domains for spin-cast P(DLLA-*co*-CL) at room temperature (white squares) and for 3-h-annealed copolymer (black squares).

Moreover, the diameters of the spherical domains in the case of the room temperature-nanostructured copolymer follow a Gaussian statistical distribution (Figure 6). As shown by Teraoka [31], given two different points, r1 and r2, the Gaussian distribution indicates a transition probability for r2 to move into a small volume around r1, justifying in our case a stable phase separation of CL-enriched spherical domains in a LA-enriched matrix. Instead, an exponential curve of third order is required in order to fit the experimental data of the 3-h-annealed copolymer, confirming the strong changes in the phase distribution of the two samples.

For longer annealing times, the nanostructured domains collapse and a disordered homogeneous structure is formed (not shown). It turns out that the phase segregation is characterized by a non-equilibrium geometrical rearrangement of the interfaces which tends to aggregate, minimizing the surface energy, and evolve to a dissolution of the nanostructured domains in the PDLLA-rich phase.

## Conclusions

A P(DLLA-*co*-CL) copolymer has been studied in terms of chemical structure and morphological behavior. In particular, we demonstrated the random architecture of the copolymer with a LA/CL mole ratio of 89.8:10.2 with a number average molecular weight of 28,200 g/mol. From the morphological point of view, interesting nanostructured spherical domains have been obtained representing CL-enriched spheres with an average diameter of 48 nm. The annealing treatment enlarged progressively the CL-enriched domains, maintaining their spherical shape until they collapse and a homogeneous disordered structure is obtained. This is the first time that the nanostructure of random PDLLA/PCL copolymers is revealed, representing one of few experimental evidences on the possible nanostructuration of random copolymers.

## Competing interests

The authors declare that they have no competing interests.

## Authors' contributions

The work presented here has been developed in strong collaboration between the three authors. LP and AMF carried out the experimental part, participated in the discussion with JMK, and drafted the manuscript. JMK coordinated the group and revised the final manuscript. All authors read and approved the final manuscript.
